# Mitochondrial genome datasets for the sweetpotato weevil, *Cylas formicarius elegantulus* (Coleoptera: Brentidae), collected in the United States

**DOI:** 10.1016/j.dib.2023.109432

**Published:** 2023-07-21

**Authors:** Sharon A. Andreason, Zachary Lahey, Dongyan Zhao, Katherine Mejia-Guerra, Livy H. Williams, Moira Sheehan, Alvin M. Simmons, Phillip A. Wadl

**Affiliations:** a United States Department of Agriculture, Agricultural Research Service, U.S. Vegetable Laboratory, 2700 Savannah Hwy., Charleston, SC 29414, United States of America; b Breeding Insight, Cornell University, 525 Tower Rd., Ithaca, NY 14853, United States of America

**Keywords:** Mitogenome, mtDNA, Assembly, Annotation, Coleoptera, Brentidae

## Abstract

The sweetpotato weevil, *Cylas formicarius elegantulus* (Summers) (Coleoptera: Brentidae), is one of the most destructive pests of sweetpotato worldwide. Genomic analyses of sweetpotato weevils can provide insights into their genetic diversity, population structure, and dispersal as well as provide information to support management strategies. Adult sweetpotato weevils were collected by various methods from *Ipomoea batatas* L. (sweetpotato) or *I. coccinea* L. (red morning glory) in the U.S. states of Georgia, Hawaii, South Carolina, and Texas. Genomic DNA was extracted from individual weevil specimens and sequenced using Illumina NovaSeq. A total of 181 GB of 150 base pair (bp) paired-end reads were generated for 40 specimens. Mitochondrial genomes were assembled for each specimen via reference mapping and annotated using Geneious Prime. Full mitochondrial genome sequences range from 17,141 to 17,152 bp with an average GC content of 21.8% and average coverage of 3307 × . A maximum likelihood phylogenetic analysis considering the mitochondrial protein coding genes is provided. Mitochondrial genomes and assembled reads are deposited in NCBI GenBank, providing 40 mitogenomes of *C. formicarius elegantulus* collected in the U.S.

**Specifications Table**

**Table:** 

Subject	Biological Sciences
Specific subject area	Entomology and Insect Science
Type of data	Raw sequencing reads, Assemblies, Annotations, Tables, Figures
How the data were acquired	The data were acquired from whole-body insect specimen genomic DNA extraction followed by whole-genome skim sequencing using Illumina NovaSeq 6000. Insect mitochondrial genome data and filtered SRA datasets were obtained by mapping paired-end reads to a reference genome available in GenBank using Geneious Prime.
Data format	Raw, filtered, and analyzed
Description of data collection	Sweetpotato weevil specimens were collected from *Ipomoea batatas* or *I. coccinea* by hand or pheromone-baited pitfall trap collection. Genomic DNA was extracted from individual whole-body adult insect specimens. DNA purity and concentration were measured before sequencing. Sequences were obtained by the Illumina NovaSeq 6000 followed by reference-guided assembly using Geneious Prime.
Data source location	Sweetpotato weevil specimens were collected in sweetpotato fields or greenhouses in Georgia (Tifton); Hawaii (Akaka Falls and Onomea); South Carolina (Charleston), and Texas (Cherokee County), USA.
Data accessibility	Raw sequence data, assembled mitochondrial genomes, and genome annotations are available in NCBI GenBank. Repository name: NCBI GenBank Data identification number: BioProject PRJNA945076; Accessions OQ763214–OQ763253 Direct URL to data: Cylas formicarius (ID 945076) – BioProject – NCBI (nih.gov)

## Value of the Data

• These data are useful for analysis of intraspecific divergence within the mitochondrial genomes of sweetpotato weevils.
• These mitochondrial genome sequences will be a useful resource for entomologists and pest management professionals seeking to analyze and determine the genetic differences among sweetpotato weevil populations.
• The data can be used to identify SNPs and other genetic markers to discriminate weevil populations, to study phylogenetic relationships among weevil populations, to determine region of origin, and to develop sequence-based weevil identification tools.

## 1 Objective

Adult *Cylas formicarius elegantulus* were collected for genome sequencing and analysis of genetic diversity and population structure of weevils captured across a wide swath of the U.S. sweetpotato production areas. Mitochondrial genomes were assembled for the purpose of providing the first mitochondrial genomes for sweetpotato weevils collected in the U.S., for determining intraspecific sequence divergence within mitochondrial genes, and for inferring phylogenetic relationships among sweetpotato weevil populations.

## 2 Data Description

**Fig. 1:**
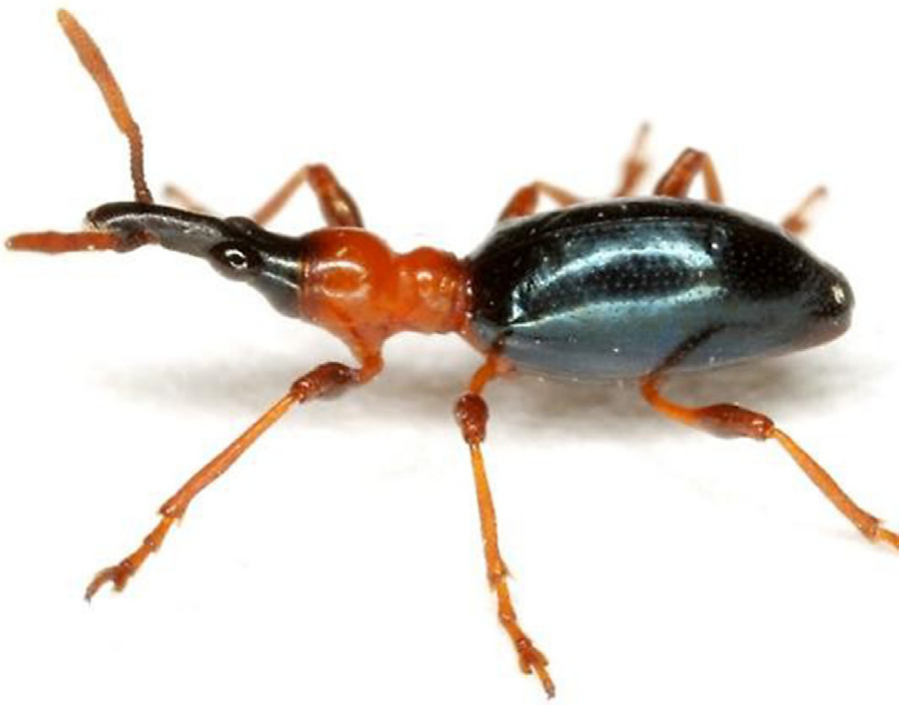
Adult *Cylas formicarius elegantulus*. Photo credit: Mike Quinn, TexasEnto.net.

**Fig. 2:**
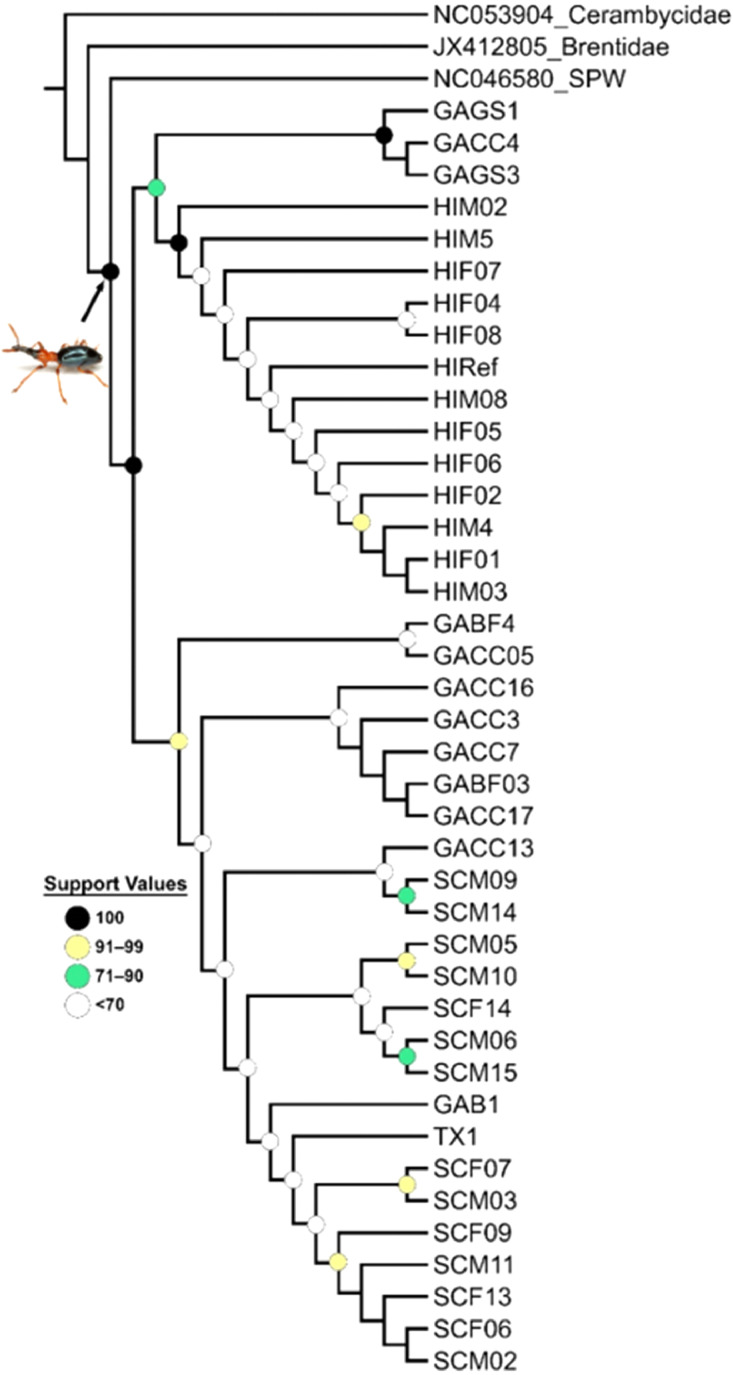
Maximum Likelihood phylogenetic analysis of *Cylas formicarius elegantulus* mitochondrial protein-coding genes (nucleotides). Support values are indicated by colored circles placed at nodes. Nodes without support values within a specific clade represent identical sequences (e.g., HIM4, HIF01, HIM03). *Cylas formicarius elegantulus* photo credit: Mike Quinn, TexasEnto.net.

Skim sequencing was performed on *C. formicarius elegantulus* (Fig. 1) genomic DNA to generate genomic sequences for population genetics studies. Weevil specimen and collection details are summarized in Table 1. Mitochondrial genome sequences were assembled in Geneious Prime and are available in NCBI Genbank at Accessions OQ763214–OQ763253. Assembly results are summarized in Table 2. Corresponding mapped reads are available as SRA datasets under BioProject PRJNA945076: Sweetpotato weevil genomics [1]. Mitogenomes were annotated using Geneious Prime. A maximum likelihood (ML) phylogenetic analysis was performed on the mitochondrial protein coding genes (Fig. 2).

**Table 1:** *Cylas formicarius elegantulus* specimens analyzed in this study.

Specimen	Location	Collection	Host	Sex
GAB1	Tifton, GA	Pheromone baited pitfall trap	*Ipomoea batatas*	Male
GABF03	Tifton, GA	Pheromone baited pitfall trap	*Ipomoea batatas*	Male
GABF4	Tifton, GA	Pheromone baited pitfall trap	*Ipomoea batatas*	Male
GACC05	Tifton, GA	Pheromone baited pitfall trap	*Ipomoea batatas*	Male
GACC13	Tifton, GA	Pheromone baited pitfall trap	*Ipomoea batatas*	Male
GACC16	Tifton, GA	Pheromone baited pitfall trap	*Ipomoea batatas*	Male
GACC17	Tifton, GA	Pheromone baited pitfall trap	*Ipomoea batatas*	Male
GACC3	Tifton, GA	Pheromone baited pitfall trap	*Ipomoea batatas*	Male
GACC4	Tifton, GA	Pheromone baited pitfall trap	*Ipomoea batatas*	Male
GACC7	Tifton, GA	Pheromone baited pitfall trap	*Ipomoea batatas*	Male
GAGS1	Tifton, GA	Pheromone baited pitfall trap	*Ipomoea coccinea*	Male
GAGS3	Tifton, GA	Pheromone baited pitfall trap	*Ipomoea coccinea*	Male
HI_Ref	Hawaii	AgPest 100 reference specimen	*Ipomoea batatas*	Female
HIF01	Akaka Falls, HI	Hand-catch (field)	*Ipomoea batatas*	Female
HIF02	Akaka Falls, HI	Hand-catch (field)	*Ipomoea batatas*	Female
HIF04	Akaka Falls, HI	Hand-catch (field)	*Ipomoea batatas*	Female
HIF05	Onomea, HI	Hand-catch (field)	*Ipomoea batatas*	Female
HIF06	Onomea, HI	Hand-catch (field)	*Ipomoea batatas*	Female
HIF07	Onomea, HI	Hand-catch (field)	*Ipomoea batatas*	Female
HIF08	Onomea, HI	Hand-catch (field)	*Ipomoea batatas*	Female
HIM02	Akaka Falls, HI	Hand-catch (field)	*Ipomoea batatas*	Male
HIM03	Akaka Falls, HI	Hand-catch (field)	*Ipomoea batatas*	Male
HIM4	Akaka Falls, HI	Hand-catch (field)	*Ipomoea batatas*	Male
HIM5	Onomea, HI	Hand-catch (field)	*Ipomoea batatas*	Male
HIM08	Onomea, HI	Hand-catch (field)	*Ipomoea batatas*	Male
SCF13	Charleston, SC	Hand-catch (colony)	*Ipomoea batatas*	Female
SCF14	Charleston, SC	Hand-catch (colony)	*Ipomoea batatas*	Female
SCF06	Charleston, SC	Hand-catch (colony)	*Ipomoea batatas*	Female
SCF07	Charleston, SC	Hand-catch (colony)	*Ipomoea batatas*	Female
SC0F9	Charleston, SC	Hand-catch (colony)	*Ipomoea batatas*	Female
SCM10	Charleston, SC	Hand-catch (colony)	*Ipomoea batatas*	Male
SCM11	Charleston, SC	Hand-catch (colony)	*Ipomoea batatas*	Male
SCM14	Charleston, SC	Hand-catch (colony)	*Ipomoea batatas*	Male
SCM15	Charleston, SC	Hand-catch (colony)	*Ipomoea batatas*	Male
SCM02	Charleston, SC	Hand-catch (colony)	*Ipomoea batatas*	Male
SCM03	Charleston, SC	Hand-catch (colony)	*Ipomoea batatas*	Male
SCM05	Charleston, SC	Hand-catch (colony)	*Ipomoea batatas*	Male
SCM06	Charleston, SC	Hand-catch (colony)	*Ipomoea batatas*	Male
SCM09	Charleston, SC	Hand-catch (colony)	*Ipomoea batatas*	Male
TX1	Cherokee County, TX	Hand-catch (greenhouse)	*Ipomoea batatas*	Female

**Table 2:** *Cylas formicarius elegantulus* mitochondrial genome assemblies.

Specimen	Mitogenome Accession #	Sequence Length (bp)	SRA Accession #	# Mapped Reads	Average Coverage	% GC
GAB1	OQ763214	17,149	SRX19730765	90,031	787	21.8
GABF03	OQ763215	17,152	SRX19740992	102,315	895	21.8
GABF4	OQ763216	17,147	SRX19740993	97,074	849	21.8
GACC05	OQ763219	17,146	SRX19741004	769,202	6729	21.8
GACC13	OQ763221	17,147	SRX19741015	605,513	5297	21.8
GACC16	OQ763222	17,147	SRX19741025	532,052	4654	21.8
GACC17	OQ763223	17,147	SRX19741026	715,022	6255	21.8
GACC3	OQ763217	17,146	SRX19741027	89,879	786	21.8
GACC4	OQ763218	17,146	SRX19741028	180,156	1576	21.9
GACC7	OQ763220	17,146	SRX19741029	703,276	6153	21.8
GAGS1	OQ763224	17,148	SRX19741030	539,348	4718	21.9
GAGS3	OQ763225	17,148	SRX19740994	417,995	3656	21.9
HI_Ref	OQ763238	17,145	SRX19740995	552,278	4832	21.9
HIF01	OQ763226	17,146	SRX19740996	262,776	2299	21.9
HIF02	OQ763227	17,145	SRX19740997	359,207	3143	21.9
HIF04	OQ763228	17,144	SRX19740998	254,933	2231	21.9
HIF05	OQ763229	17,141	SRX19740999	339,006	2967	21.9
HIF06	OQ763230	17,144	SRX19741000	288,496	2524	21.9
HIF07	OQ763231	17,146	SRX19741001	412,562	3609	21.9
HIF08	OQ763232	17,146	SRX19741002	354,908	3105	21.9
HIM02	OQ763233	17,146	SRX19741003	544,098	4760	21.9
HIM03	OQ763234	17,147	SRX19741005	694,717	6077	21.9
HIM4	OQ763235	17,146	SRX19741006	379,117	3317	21.9
HIM5	OQ763236	17,149	SRX19741007	694,703	6076	21.9
HIM08	OQ763237	17,146	SRX19741008	348,555	3049	21.9
SCF13	OQ763242	17,148	SRX19741009	231,530	2025	21.8
SCF14	OQ763243	17,149	SRX19741010	166,687	1458	21.8
SCF06	OQ763239	17,148	SRX19741011	193,265	1691	21.8
SCF07	OQ763240	17,144	SRX19741012	224,505	1964	21.8
SCF09	OQ763241	17,149	SRX19741013	188,219	1646	21.8
SCM10	OQ763249	17,150	SRX19741014	462,114	4042	21.8
SCM11	OQ763250	17,148	SRX19741016	293,155	2564	21.8
SCM14	OQ763251	17,148	SRX19741017	387,807	3392	21.8
SCM15	OQ763252	17,144	SRX19741018	342,605	2998	21.8
SCM02	OQ763244	17,148	SRX19741019	484,612	4239	21.8
SCM03	OQ763245	17,146	SRX19741020	605,989	5301	21.8
SCM05	OQ763246	17,144	SRX19741021	317,974	2782	21.8
SCM06	OQ763247	17,146	SRX19741022	130,730	1144	21.8
SCM09	OQ763248	17,148	SRX19741023	540,294	4726	21.8
TX1	OQ763253	17,148	SRX19741024	223,843	1958	21.8

## 3 Experimental Design, Materials and Methods

### 3.1 Specimen Collection, DNA Extraction, and Sequencing

Adult *C. formicarius elegantulus* were collected from locations in the U.S. states of Georgia, Hawaii, South Carolina, and Texas by various methods detailed in Table 1. Genomic DNA was extracted from individual whole-body male and female sweetpotato weevils with the DNeasy Plant Mini Kit (Qiagen, Venlo, Netherlands) with modifications [2]. DNA quantity was measured on a NanoDrop 2000 spectrophotometer (ThermoFisher Scientific, Waltham, MA, USA). Whole-genome skim sequencing was done using Illumina NovaSeq 6000 at Novogene (www.novogene.com).

### 3.2 Mitochondrial Genome Assembly and Annotation

Mitochondrial genomes of 40 sweetpotato weevils were assembled in Geneious Prime version 2022.0.2 using the Map to Reference tool with the *C. formicarius* complete mitochondrial genome assembly from China as a reference (NCBI Reference Sequence: NC_046580.1; [3]). The Geneious mapper was set at medium sensitivity and five iterations assembled the fastq paired-end sequence datasets. Assemblies were circularized by trimming overlapping ends. Complete mitogenomes and assembled read datasets were deposited in GenBank (BioProject PRJNA945076). Mitogenome sequences were annotated using NC_046580.1 as a reference and the ‘Annotate from Database’ feature in Geneious Prime. Annotations are available in GenBank.

### 3.3 Phylogenetic Analysis

Phylogenetic analyses were conducted under an ML framework in IQ-TREE (v.2.1.3) [4]. The nucleotide sequences for each of the 13 mitochondrial protein coding genes in 43 taxa were aligned with MAFFT (v.7.249) [5], and the best nucleotide substitution model for each gene was selected with ModelFinder [6]. Branch support was estimated with 1000 ultrafast bootstrap replicates [7]. Ten independent tree searches were performed, and the tree with the greatest log-likelihood score was taken as the ML tree (Fig. 2).

## Ethics Statements

The work involving insect invertebrates detailed herein complied with ARRIVE guidelines and the National Institutes of Health guide for the care and use of laboratory animals (NIH Publications No. 8023, revised 1978).

## CRediT authorship contribution statement

**Sharon A. Andreason:** Conceptualization, Methodology, Formal analysis, Data curation, Visualization, Writing – original draft. **Zachary Lahey:** Methodology, Software, Formal analysis, Visualization, Writing – review & editing. **Dongyan Zhao:** Conceptualization, Methodology, Formal analysis. **Katherine Mejia-Guerra:** Conceptualization, Methodology, Formal analysis. **Livy H. Williams:** Resources, Writing – review & editing. **Moira Sheehan:** Conceptualization, Methodology, Writing – review & editing. **Alvin M. Simmons:** Resources, Writing – review & editing. **Phillip A. Wadl:** Conceptualization, Resources, Writing – review & editing.

## Data Availability

Sweetpotato Weevil Mitochondrial Genomes (Original data) (GenBank).
